# Addressing Mental Health in Rural Settings: A Narrative Review of Blueberry Supplementation as a Natural Intervention

**DOI:** 10.3390/nu16203539

**Published:** 2024-10-18

**Authors:** Katy E. Venable, Charles C. Lee, Joseph Francis

**Affiliations:** Department of Comparative Biomedical Sciences, School of Veterinary Medicine, Louisiana State University, Baton Rouge, LA 70803, USA; cclee@lsu.edu (C.C.L.); jfrancis@lsu.edu (J.F.)

**Keywords:** depression, anxiety, major depressive disorder, generalized anxiety disorder, rural health, blueberry, nutrition, food supplementation

## Abstract

Depression and anxiety are major public health issues; however, there is an unmet need for novel, effective, and accessible treatments, particularly in rural communities. Blueberries are an unexplored nutraceutical for these conditions due to their excellent nutritional profile, with particularly high levels of polyphenols and anthocyanins and benefits on mood, cognition, and health. Here, we present a narrative review of the literature concerning the etiology and treatments of major depressive disorder (MDD) and generalized anxiety disorder (GAD). In both animal and human studies, blueberry supplementation can ameliorate behavioral symptoms of both anxiety and depression. The mechanistic underpinnings of these behavioral improvements are not fully defined, but likely involve biochemical alterations in the gut–brain axis, including to inflammatory cytokines, reactive oxygen species, and growth factors. We also review the limitations of traditional therapies in rural settings. Finally, we assess the potential benefit of nutraceutical interventions, particularly blueberries, as novel therapeutics for these distinct, yet related mental health issues.

## 1. Introduction

Depression and anxiety disorders are common, costly, and major public health concerns. According to the National Institute of Mental Health (NIMH), major depressive disorder (MDD) and anxiety disorders affected 8.3% and 19.1% of the American population in 2021, respectively [1,2]. MDD and anxiety both affect women more than men; in 2021, 10.3% of females experienced a major depressive episode compared to 6.2% of males, while a striking 23.4% of females were diagnosed with anxiety compared to 14.3% of males [1,2]. Globally, the COVID-19 pandemic increased depression rates by 27.6% and anxiety by 25.6% in 2020, increasing more in women than men and younger age groups than older [3]. The Global Burden of Diseases, Injuries, and Risk Factors Study has consistently found that depression and anxiety have contributed to the global burden of disease for the past 30 years, both being among the top 20 leading causes of disability among all age groups and sexes, but accounting for the second and third leading causes of disabilities among women aged 19–50, and in the top 10 for both sexes aged 9–50 [4]. Depression and anxiety are often comorbid; 60% of people diagnosed with anxiety are also diagnosed with MDD, while 45.7% of those with depression will also experience an anxiety disorder at some point in their life. Those with comorbid anxiety and depression are more likely to commit suicide and have worse overall prognoses and health outcomes [5,6,7,8].

The high levels of depression and anxiety observed globally are particularly alarming when also considering comorbidities and the increased risk of developing other health conditions such as cardiovascular diseases [9,10,11], diabetes [12], cancer [13], substance abuse disorders [14], obesity, metabolic disorders [15], and Alzheimer’s disease [16,17,18,19]. These diseases are also among the top leading causes of death and disability in the US and globally [4].

Importantly, depression and anxiety appear to have bi-directional relationships with other diseases. Depression or anxiety increases the risk of developing chronic diseases and diseases of aging [20]. Conversely, serious or chronic health condition(s) also increase the risk of developing depression and anxiety disorders [21]. For instance, cancer diagnoses, chemo treatments [22], hemodialysis, multiple sclerosis (MS) [23], and cardiovascular disease all increase depressive symptoms, while rheumatoid arthritis and hepatitis [24,25] increase symptoms of anxiety. Finally, depression and anxiety can affect the management of chronic diseases, negatively impact outcomes, and increase mortality in chronic diseases [26]. Dysregulated systemic and/or cellular metabolism or mitochondrial function may be at the root of mental health disorders, and mediate their bi-directional comorbidities with each other and other chronic inflammatory, neuroendocrine, and cardiometabolic health conditions, as reviewed more fully elsewhere [27,28,29].

## 2. MDD and GAD Exhibit Substantial Symptom Overlap

The Diagnostic and Statistical Manual of Mental Disorders, Fifth Edition (DSM-5) describes, “Depressive Disorders” and “Anxiety Disorders” as general categories of disorders that share features, with substantial overlap within and between categories [30,31]. Here, we focus on major depressive disorder (MDD) and generalized anxiety disorder (GAD), which exhibit between 40% and 80% comorbidity. MDD is characterized primarily by a “loss of interest or pleasure” lasting more than 2 weeks, while GAD is characterized primarily by “excessive anxiety or worry” lasting more than six months. However, each diagnosis requires other accompanying somatic and cognitive symptoms, such as tension, restlessness, sleep and appetite dysregulation, and trouble with concentration, attention, and memory (Table 1).

Many symptoms of MDD and GAD, particularly the somatic and bio-energetic symptoms (fatigue, sleep and appetite issues, and weight gain/loss) may be indicative of metabolic dysfunction [32,33], while other symptoms like heart palpitations and hyperventilation indicate autonomic nervous system dysfunction and a lack of balance between sympathetic and parasympathetic activity [34,35]. Indeed, metabolic syndrome, a cluster of cardiometabolic risk factors including chronically elevated c-reactive protein (CRP), dyslipidemia, hypertension, and hyperglycemia, is more common in MDD and psychiatric disorders generally than in the general population [33]. Likewise, reduced autonomic flexibility and dysregulated autonomic function are apparent in both MDD and GAD [36,37]. Altogether, while categorically distinct in their core symptom profiles, there is clearly substantial symptomatic and mechanistic overlap between MDD and GAD.

The potential metabolic roots of MDD and GAD are helpful to consider when examining their range of symptoms, which present variably in different people. For example, some individuals might present with a suppressed appetite, while others may present with an increased appetite; some individuals might present with insomnia, while others present with hypersomnia; and some people might feel heavy and leaden, while others are restless and hyperactive. Although these appear as distinct symptoms, Table 1 demonstrates how symptoms of both anxiety and depression can essentially be collapsed into a few categories that share features. Further, Caspi et al. (2014) identified significant symptom overlap across DSM categories and found that psychiatric disorder risks and outcomes are best characterized by a general psychopathology factor (p factor) [31]. Although there is substantial overlap, the main differences lie in the affective symptoms, i.e., depression is associated with feelings of depression (loss of interest or pleasure or feelings of emptiness, guilt, or worthlessness) while anxiety is associated with feelings of worry or panic. Ultimately, symptoms of MDD and/or GAD converge and lead to dysfunction or a loss of function in one or more domains of a person’s life including their social life/relationships, school or work, personal care, health, etc. This dysfunction or loss of function may reflect metabolic disturbances.

The symptoms of MDD and GAD may have a bi-directional causal relationship to their known physiological and metabolic correlates. MDD and GAD are associated with dysregulation of the neuroendocrine system, particularly the hypothalamic–pituitary–adrenal (HPA) axis [17,38,39] and the immune [40,41], cardiovascular [35,42,43,44], autonomic, and neuromuscular systems [16,17,34]. Again, these relationships can be bi-directional, similar to that observed in sickness behavior, in which illness or pain can drive depressive symptoms by potentially increasing pro-inflammatory cytokine expression in both the periphery and CNS [45]. Likewise, dysregulation of autonomic function can drive anxiety symptoms [46]. Further, dysbiosis of the gut microbiome and dysregulation of the gastrointestinal system are observed in both MDD and GAD [47,48], and targeting the microbiome may be effective at alleviating symptoms [49,50,51,52,53]. Overall, MDD and GAD are associated with systemic neuroendocrine, cardiometabolic, gastrointestinal, and immune dysfunction.

The affective and cognitive features of MDD and GAD have a complex relationship with somatic and bioenergetic symptoms, promoting behaviors that perpetuate their psychopathological states. In fact, those with MDD and/or GAD are more likely to engage in addictive behaviors like smoking cigarettes and drinking alcohol [54,55,56,57], which in turn results in a higher risk for developing MDD and GAD [58]. These attempts at self-medication acutely alleviate symptoms, but have negative physiological and psychological consequences, especially with chronic, long-term use. Anxiety and the associated increased threat sensitivity in MDD and GAD are also associated with a low-grade inflammatory state, which also likely contributes to physiological comorbidities and disease symptoms [59]. Together, these behaviors contribute to the observed accelerated aging and the increased risk of diseases of aging [60,61] in those with psychiatric diagnoses, particularly MDD and GAD. This concept has been introduced as disease progression, or neuroprogression in the brain [38], which further highlights the need for effective treatments for MDD and GAD.

## 3. Novel Treatments and/or Adjuvants Are Needed

**Figure 1:**
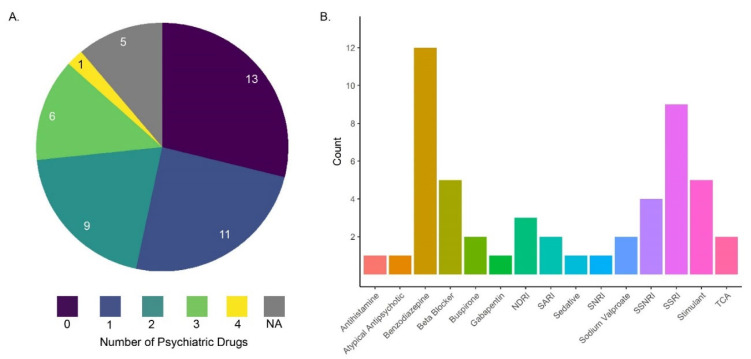
(**A**) The distribution of the number of psychiatric prescriptions that patients were prescribed simultaneously; the numbers in the pie chart are the number of patients on either 0, 1, 2, 3, or 4 psychiatric medications [64]. (**B**) Among the broad classes of drugs prescribed to patients in panel (**A**), benzodiazepines (gold bar) and selective serotonin reuptake inhibitors (SSRIs: purple bar) were most commonly used. Many other prescriptions were also prescribed in conjunction with benzodiazepines and SSRIs [64].

The goal of treatments for MDD and GAD is the full remission of symptoms and a return to the individual’s baseline functioning with no relapse. To this end, the American Psychological Association (APA) recommends psychotherapy and/or medication for the treatment of MDD [62]. Although the APA has not published official guidance for the treatment of GAD, psychotherapy and/or medication are also the first-line treatment approaches [63]. In clinical practice, pharmacotherapies are the dominant treatment strategy, particularly in rural areas, which lack access to mental health professionals and resources. Indeed, we have found in our rural clinic studies that participants are routinely prescribed several classes of psychiatric medications for depression or anxiety symptoms [64] (Figure 1).

The medications prescribed sometimes differ between MDD and GAD patients, but generally, second-generation antidepressants (i.e., selective serotonin reuptake inhibitors (SSRIs)) are prescribed in the case of MDD and comorbid MDD and anxiety disorders [5,65]. For MDD, if SSRIs are inadequate, serotonin and norepinephrine uptake inhibitors (SNRIs), monoamine oxidase inhibitors (MAOIs), or Tricyclic antidepressants are needed. GAD and anxiety disorders are commonly treated with “anxiolytics” which target either the CNS or the periphery; in the U.S., gabapentin is currently the most prescribed treatment for anxiety, followed by trazodone hydrochloride, buproprione, and alprazolam [66]. However, benzodiazepines are often the most commonly prescribed [64] (Figure 1). Antidepressants, typically SSRIs and SNRIs, are also commonly used because they are less likely to cause adverse events and side effects and create dependence compared to some common anxiolytics like benzodiazepines [67,68]. Benzodiazepines are known to cause dependence and are particularly risky since benzodiazepine withdrawal can lead to serious consequences [69]. Generally, a doctor or nurse practitioner will work with patients to determine the treatment approach to best relieve symptoms for the individual with limited adverse side effects. Overall, there are a number of drugs and drug combinations used to treat MDD and GAD.

It is important to note that while SSRIs are commonly prescribed, there is significant concern and controversy over the “serotonin hypothesis of depression” and the over-prescription of antidepressants [70]. Independent meta-analyses highlight a few key findings regarding the antidepressant literature: (1) short study designs (when most people take antidepressants chronically); (2) efficacy concerns—antidepressants are more effective than a placebo for patients with severe symptoms; however, they are not more effective than a placebo in people with mild to moderate symptoms [71]; and (3) psychotherapy alone is more effective than any pharmacotherapy alone [65].

Despite the widespread use of pharmaceuticals, psychotherapy is an effective treatment for both depressive disorders and anxiety disorders either as a standalone treatment or in combination with pharmacological approaches [72,73,74]. Direct comparisons are difficult to perform experimentally [75], although evidence suggests that psychotherapy alone is more effective than pharmacotherapy alone [76,77]. However, such therapy is less accessible than pharmaceuticals for several reasons. It is (1) time-consuming, (2) costly, (3) not readily covered by insurance, (4) motivation-dependent, and (5) difficult to find the right therapist or type of therapy [78]. So, while psychotherapy can be an effective treatment strategy, most individuals, particularly in rural settings, do not have the time, money, motivation, or access.

## 4. Disparities in Urban and Rural Health Care

Rural areas lack adequate numbers of qualified health care professionals and facilities per capita, particularly for mental health care [64,79,80,81], which contributes to worse outcomes, even in similar disease conditions [82,83]. To address the paucity of health care in rural areas, the Rural Health Clinic Services Act was passed in 1977 which aimed to increase access to primary health care services in rural, underserved areas through the creation of Rural Health Clinics (RHCs) [84]. RHCs can be public, non-profit, or for-profit, and are incentivized by enhanced reimbursement rates for federally funded Medicare and federal and state Medicaid services [85].

Interestingly, the main disparity between urban and rural mental health is not in the prevalence of disorders like depression and anxiety, but rather it is in access to suitable care [86,87]. Not only are individuals in rural areas less likely to seek treatment for mental health issues but they also have few or no options in regards to mental health care professionals, particularly specialized or highly trained professionals [82,86]. For example, in rural Marksville and Cottonport, Louisiana, where we have previously conducted studies, this was the case, and many received pharmacological treatment from their primary care physicians at RHCs, since it is all the providers had to offer in terms of treatment approaches [64]. The owners and operators of the clinics are actively seeking new, effective, and accessible mental health treatment options for their community.

Improving and modernizing Rural Health Clinics remains a challenge for increasing access, treatment, and care. Foundational legislation, such as the Rural Health Clinics Services Act of 1977, started addressing the lack of health care in rural areas, and newer legislation like the Rural Health Clinics Modernization Act aims at further enhancements of care. Preventative health care and its initiatives and incentives are other important pieces of the puzzle that could potentially have a large impact on rural health in America. Similarly, rural health research is impeded by many of these same factors, and current solutions to rural health research are often inadequate or ineffective; thus, knowing exactly what interventions or approaches to use or study and assessing how effective they are or predicting how effective they might be remains a major challenge. In this regard, the need for alternative approaches to the treatment of mental health conditions, such as MDD and GAD, are required. As such, the implementation of nutritional-based interventions represents a potentially impactful avenue in rural settings.

## 5. Blueberries as a Nutraceutical Intervention

Among the non-pharmaceutical approaches to addressing depression and anxiety, diet is a highly significant contributor approach to mental health. The ingestion of many nutrients has been linked to mood improvement, including B vitamins, vitamin D, minerals, antioxidants, and omega-3 fatty acids. Such nutrients promote the health of the gut microbiome, which can positively influence mood and reduce anxiety through the production of neurotransmitters like serotonin. Probiotic and prebiotic foods can support such a healthy gut microbiome, leading to improved mood. Among the latter class, blueberries have emerged as a potential superfood that contains many of these important nutrients.

Blueberries are a potentially accessible treatment for depression and anxiety. The fruit is native to coastal northeast North America, but cultivation, starting in the early 20th century, led to an expansion of regions that can grow and access blueberries [88]. This expansion, along with the burgeoning, positive health research that began in the early 2000s [89,90], led to dramatic increases in the consumption, import, and export of blueberries over the past 20 years; blueberries are one of the most important fruit products in the United States, with the average person consuming approximately 2.5 pounds per year [91]. Blueberries are a great candidate nutraceutical as they are generally palatable (being enjoyable for many), low risk, and have an excellent nutritional profile, containing particularly high amounts of various bioactive phytochemicals, specifically polyphenols and anthocyanins [89,92]. Some other notable macro and micronutrients are fiber, manganese, and ascorbic acid [93]. Importantly, the nutritional profile of blueberries is complex and varies widely between species [94], developmental stages [95], environmental growing conditions [96] (i.e., regional variations [97], seasonal variations, and organic versus conventional growing methods [98]), and processing [99,100,101] and storage methods [102]. This compositional complexity and variation lend to the complexity and importance of blueberries and high-quality blueberry research.

Blueberries are nutrient-dense (Table 2) [93], but “what is the *active ingredient* in blueberries that make them so good for health?” This is by far the most common question regarding their potential therapeutic use. In this regard, anthocyanins are typically identified as the dominant phytochemicals present in blueberries. These chemicals are pigmented polyphenols that give plants and their fruits characteristic red, blue, and purple hues. Polyphenols, specifically anthocyanins, garner particular attention in blueberry research, often emphasized as the “active ingredient” in blueberries because their metabolites are bioavailable (even in small amounts) [103], distribute to different tissues, including the brain [104,105,106,107,108], and are associated with a number of health benefits [109,110,111]. Specifically, anthocyanins have beneficial effects on the cardiovascular system [112,113], the gut microbiome [114,115,116], insulin sensitivity, glucose metabolism, and cholesterol [117,118]. Overall, this evidence demonstrates that anthocyanins have beneficial effects on multiple systems that affect the overall metabolic health of humans.

Why might the anthocyanins present in blueberry have these effects? As mentioned, anthocyanins are phytochemicals, a special and interesting class of micronutrients. These chemicals evolved in plants to theoretically combat environmental stressors and insults encountered in nature [119]; this (including the forms and effects of phytochemicals) was influenced by the co-evolutionary relationship that plants and animals shared over Earth’s evolutionary history [119]. Phytochemicals endow plants with presumed protective properties against environmental stressors [120] due to ultraviolet radiation from sunlight [121], water [122,123], heat, fungi [124,125], and viruses [126]. Yet, through the consumption of plants, humans might also receive some of the protective benefits of phytochemicals and their metabolic products [127,128,129]. It is also noteworthy that isolated polyphenols and/or anthocyanins sometimes do not work as well or as consistently as in combination with each other [130]. Some fractionations may be more beneficial for specific endpoints [131] or have different effects on gut microbes [132]. Further, glycosylated polyphenols and anthocyanins are more biostable when bound to pectin [133], indicating the potential importance of how other nutrients might influence phytochemical bioavailability. However, the effects of isolated phytochemicals [112,118] and whole fruit preparations have been poorly investigated in humans [132], likely due to the technical challenge of creating purified polyphenol or anthocyanin isolates [101]. So, although anthocyanins are promising compounds and their metabolites are bioactive, the effects of other phytochemicals and nutrients in blueberries cannot be ignored [130,132].

Fiber is another important and abundant nutrient in blueberries that is associated with several health benefits [93]; however, it is unexplored in the blueberry literature [89,90,134]. It is a significant source of fiber, particularly in the American diet which generally lacks adequate dietary fiber [135,136]. Interestingly, in a 2023 meta-analysis, dietary fiber was inversely associated with the risk of MDD in adults in a dose-dependent manner [137], although a similar link with anxiety could not be explored due to insufficient studies. Further, dietary fiber is correlated with an inverse risk of the same metabolic disorders associated with MDD and GAD that we have previously discussed, i.e., cardiovascular diseases [138,139], diabetes [140], and metabolic syndrome [141,142]. These relationships are potentially mediated by the microbiome–gut–brain axis and, more specifically, the production of short-chain-fatty-acids (SCFAs) which are the product of microbial fiber fermentation [143]. This information does pose the question of how the presence of fiber in blueberries influences the benefits that blueberries have on health [89,90].

Numerous mechanisms can be involved in the therapeutic actions of single-molecule pharmaceuticals, and the same is true for micro- and macronutrients that play roles in various biochemical reactions. Therefore, for a plant or plant extract containing dozens of bioactive compounds [144], the number of independent or interacting mechanisms involved is potentially massive. Our capacity to develop novel chemical structures and novel medications through medicinal chemistry lags behind the natural capacity of plants and fungi. In fact, natural products make up more than one-third of all FDA-approved medications [145]. The FDA even has a drug developmental pathway specifically for natural products [146]; FDA-approved medications have already been developed through this pathway, including the plant extracts Mytesi (crofelemer), and Veregen (sinecatechins). Importantly, unlike most FDA-approved drugs, these medicines are extracts of natural products, not synthetic compounds, and therefore contain trace amounts of other natural compounds.

This underutilized drug development pipeline allows for the development of natural product extracts for medical use in the United States (NCT00547898) [147,148]. Furthermore, an emerging body of literature suggests that plant extracts containing a host of compounds can be more effective than single isolated compounds alone [149] due to synergistic or combinatory effects. Overall, the challenge to studying natural products for medical disorders is considerably more complicated than pharmaceuticals, but this enormous investment may also produce massive rewards by deepening our understanding of the pathogenesis of common medical disorders and improving or developing novel treatment approaches.

**Table 2:** The nutrient contents of USHBC freeze-dried blueberry powder (Tifblue/Rubel 50/50 blend) with % daily intakes calculated based on a 2000 calorie diet [64,150,151,152,153,154,155]. NA, not applicable.

Nutrient	Content Per Dose (24 g)	% Daily Intake
Calories	94.6 kcal	4.70%
Protein	0.7 g	1.40%
Carbohydrates	22.0 g	8%
Fat	0.4 g	0.50%
Saturated Fat	0.1 g	0.50%
Trans Fatty Acids	0 g	0%
Total Sugars	16.9 g	33.80%
Fructose	8.6 g	NA
Glucose	8.2 g	NA
Sucrose	0.002 g	NA
Maltose	0.1 g	NA
Lactose	0.02 g	NA
Dietary Fiber	5.4 g	19.30%
Insoluble Fiber	4.2 g	NA
Soluble Fiber	1.4 g	NA
Cholesterol	0 mg	0%
Total Beta Carotene	1.4 µg RAE	NA
Vitamin C	4.1 mg	4.50%
Calcium	10.2 mg	0.90%
Iron	0.2 mg	1.10%
Potassium	114.7 mg	2.40%
Sodium	0.7 mg	0.03%
**ORAC**	12,360 µmole TE	NA
**Phenolics**	744 mg	NA
**Anthocyanins**	254.4 mg	NA

In this regard, blueberries that we and others have studied contain several nutrients, phenolics, and anthocyanins (Table 2) [64,150,151,152,153,154,155]. One reported measure, the Oxygen Radical Absorbance Capacity (ORAC) is an in vitro measure of antioxidant capacity that measures a food’s ability to inhibit the production of reactive oxygen species (ROS) [156,157]. A higher ORAC was previously implicated as translating into clinical applications for preventing or treating disease [156,157]. However, this approach failed to reliably translate into consistent and coherent physiological findings in vivo, leading the USDA to withdraw their ORAC database and diminished attention on ORAC values [158,159].

## 6. Mechanism of Action

When considering the mechanisms by which blueberries exert effects on human mood and behavior, it is useful to assess the relevant blueberry literature according to preclinical, in vitro, and in vivo animal studies, and human clinical studies. Although there is in vitro evidence that blueberries are antioxidant and anti-inflammatory, in vitro studies are the most limited since they generally assess the effects of blueberry compounds as they exist in the fruits or their preparations. While interesting, the results do not always translate to expected clinical outcomes and conclusions about their in vivo effects. Since the route of administration for blueberries is the digestive system, blueberry compounds directly interact with the gut microbiome and cells, with the metabolized products entering systemic circulation [103]. Felgus-Lavefve et al. (2022) offer an excellent review of the anti-inflammatory and antioxidant effects of blueberry in in vitro models [160].

In vivo studies using animal model systems have clearly indicated a role for the therapeutic effects of blueberries in ameliorating stress-related behaviors. In a previous study from our group, a 2% blueberry diet was administered to rats either prior to or after they were subjected to predator exposure and psychosocial stress [150], a model of posttraumatic stress disorder (PTSD). The stress model increased anxiety-like behaviors measured by the elevated plus maze, and also elevated reactive oxygen species (ROS) production, norepinephrine (NE), IL-1β, IL18, TLR4, and HMGB1, and reduced serotonin (5-HT) and IL-4 in the hippocampus and pre-frontal cortex of rats. Pretreatment with a 2% blueberry enriched diet prevented these effects [150], and did so without increasing NE levels like the SSRI sertraline [151].

In addition, blueberries reduced stress in LPS-induced depression models in rodents [161,162]. For example, pretreatment with blueberry extract (100 or 200 mg/kg) for 7 days prevented many LPS-induced depressive-like effects including those on (1) behavior, as measured via tail suspension and splash tests, (2) increased TNF-α and oxidative stress in the cortex, hippocampus, and striatum to a similar extent as 20 mg/kg fluoxetine (an SSRI commonly used to treat depression), and (3) increased serum ROS [161,162]. These findings were replicated and expanded to demonstrate that 200 mg/kg blueberry extract (via an ethanol–water vehicle) prevented other LPS-induced depressive-like effects, such as (1) behavior measured via a forced swim test in mice, (2) a decrease in Na+/K+-ATPase and acetylcholinesterase (AChE) activity, (3) a decrease in MAO-A in the hippocampus, and (4) the upregulation of TNF-α, IL-1β, and IL-10 transcripts in the cortex [163]. This study also demonstrated evidence that malvidin glucosides might interact with Indoleamine 2,3-dioxygenase (IDO), the rate-limiting enzyme of tryptophan metabolism [164]. Finally, serum extracted from participants supplemented with blueberry showed reduced nitrites, iNOS, COX-2, and TNF-α in LPS-treated microglia derived from rats compared to serum from control participants [165].

Blueberry treatment is also beneficial in the ketamine-induced manic depression model in rats [166]. In these studies, pretreatment with blueberry (200 mg/kg, 14 days) reduced ketamine-induced elevation of serum IL-6, hyperlocomotion, and oxidative stress in the cortex, striatum, and hippocampus, similar to pretreatment with 25 mg/kg lithium [166]. Another study found that ketamine-induced mania increased ROS, lipid peroxidation, nitrite, AChE, and Na+, K+-ATPase activity, and decreased antioxidant enzymes in the cortex and hippocampus of rats, and that these effects were prevented with a pretreatment of blueberry, similar to lithium as a positive control [167]. These results are corroborated by another study that determined 300 mg/kg to be an optimal dose of a methanol blueberry extract to ameliorate depressive-like behaviors, measured by the tail-suspension test in a chronic mild stress model in mice; further, this study investigated an in vitro digestion model which demonstrated chlorogenic acid, ferulic acid, catechin, epicatechin, quercetin, and malvidin as bioavailable compounds from blueberries [168]. Blackberry juice, which has similar high levels of phytochemicals as blueberries, administered to rats subjected to chronic mild stress, ameliorated anxiety-like behavior to a similar extent as a benzodiazepine in the elevated plus maze and depressive-like behavior measured via a forced swim test; the moderate dose of 5.83 mg/kg of anthocyanins and 27.10 mg/kg of polyphenols used in this study appeared to be most effective [169]. Finally, animal studies have also demonstrated the positive effects of blueberries for measures of cognition, memory, and plasticity, and that these effects were accompanied by increases in glutathione peroxidase, ascorbic acid, CREB, ERK1/2 signaling, and BDNF [170,171,172].

A few prior studies have evaluated and demonstrated the positive effects of blueberry on mood or symptoms of depression and/or anxiety in limited populations such as healthy adolescents (13 g of freeze-dried whole blueberry powder (WBB) for 4 wks) [173], children and young adults (30 g of WBB once per test day) [174], post-partum women (blueberry extract and juice on postpartum days 4–5) [175], and healthy adults (blueberry juice 60 mL/day for 20 days) [176]. These studies used a wide range of conditions to demonstrate the positive effects on behavior and mood following blueberry consumption. For instance, in the study of adolescents and young adults, whole blueberry powder (WBB) supplementation was provided briefly before testing for behavioral affect, which was found to improve [174]. Similarly, the study in healthy adolescents used a longer dosing of WBB supplementation which resulted in improvements in mood and affect [173]. In healthy adults, the placebo-controlled study demonstrated beneficial effects on depression and anxiety measured via the Beck Depression Inventory and State Trait Anxiety Inventory, along with improvements in total cholesterol and LDL levels [176]. Finally, the study in women in the post-partum period was an open-label study, in which women were supplemented with a combination of 2 g tryptophan, 10 g tyrosine, and blueberry juice with blueberry extract over day 3, 4, and 5 postpartum or received no supplement (control). On postpartum day 5, depression symptoms were evaluated via the visual analog scale after a sad mood induction procedure (MIP); the MIP induced a depressed mood in the control group, but not the supplemented group [175].

Some clinical studies have implied beneficial effects on executive function, cognition, and memory in various age groups and conditions from the acute and long-term administration of blueberry [177,178,179,180,181,182]. For instance, in a special journal issue, Cheng et al. (2024) [183] demonstrated the positive effects of wild blueberry extract on circadian-mediated cognitive performance and cardiovascular health. However, it is important to note that task-specific benefits sometimes differ between studies [183]. Some studies have demonstrated that these effects are accompanied by improved metabolic features, like decreased insulin resistance/glucose responses [184] and vascular function, [185] and enhanced neural activation [186] or cerebral blood flow (CBF), [187], while others found no effects on CBF, despite observing other positive vascular effects, like lowered systolic blood pressure (BP) [188]. Cognitive benefits have also been suggested to follow 90 days of blueberry supplementation and are correlated with levels of specific blueberry metabolites in plasma [189]. Urinary levels of flavanol metabolites may also be correlated with the effects of cognitive enhancement [190].

**Figure 2:**
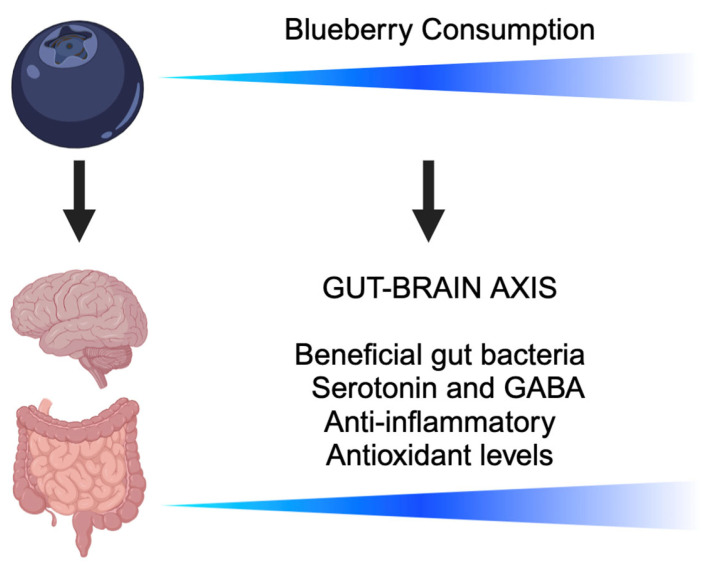
A schematic illustrating the benefits of increased blueberry consumption along the gut–brain axis. Blueberry supplementation is associated with promoting beneficial gut bacteria, increasing the production of mood-related neurotransmitters such as serotonin and gamma-amino butyric acid (GABA), decreasing the level of inflammatory cytokines, and increasing antioxidant levels [103,104,105,106,107,108,109,110,111,112,113,114,115,116,117,118].

Finally, blueberries likely mediate their positive effects through changes to the gut microbiome, which supports many physiological processes, including mental health [103,104,105,106,107,108,109,110,111,112,113,114,115,116,117,118]. As noted above, the gut microbiome can influence brain function and mood through multiple pathways, including immunomodulation, neurotransmitter production, and gut barrier function [103,104,105,106,107,108,109,110,111,112,113,114,115,116,117,118] (Figure 2). The polyphenols in blueberries, such as anthocyanins and flavonoids, positively influence gut microbiome composition, acting as prebiotics to promote the growth of beneficial bacteria while inhibiting pathogenic bacteria, resulting in an overall reduced risk of developing MDD and GAD [103,104,105,106,107,108,109,110,111,112,113,114,115,116,117,118] (Figure 2). In addition, these polyphenols may enhance gut barrier integrity, reducing systemic inflammation and positively influencing mental health [103,104,105,106,107,108,109,110,111,112,113,114,115,116,117,118]. Nevertheless, the impact of blueberries on the gut microbiome in improving mood disorders still requires additional research to identify their most effective mechanisms.

## 7. Conclusions

Overall, prior studies strongly indicate that blueberries have beneficial effects on various aspects of health. Blueberries can positively affect the cardiovascular system, the gut microbiome, the immune system, glucose and lipid metabolism, and the CNS. The positive effects of blueberries may act primarily through the periphery, particularly in the gut microbiome, the cardiovascular system, and energy metabolism. These effects might contribute to their positive effects on cognition, mood, and behavior. Importantly for rural settings, blueberries can be readily implemented as a nutraceutical approach for conditions such as GAD and MDD. As such, their substantial prior justification warrants further investigation of blueberry in the treatment of depression and anxiety in human populations, particularly in rural settings.

## Figures and Tables

**Table 1:** Symptoms of major depressive disorder (MDD) and generalized anxiety disorder (GAD).

	MDD [30]	GAD [30]
**Affective**	Depressed mood or loss of interest or pleasure. Feelings of emptiness. Psychomotor agitation. Feelings of worthlessness. Excessive/inappropriate guilt. Irritability.	Excessive anxiety or worry. Irritability. Sense of impending danger or doom. Panic.
**Cognitive**	Difficulty paying attention or concentrating. Indecisiveness. Impaired memory.	Difficulty concentrating. Mind going blank.
**Somatic**	Psychomotor agitation. Feeling heavy or like there is lead in the body. Pain (ex—back pain, headaches). Restlessness.	Restlessness. Muscle tension. Heart palpitations/racing. Nausea/gastric issues. Hyperventilation.
**Cognitive**	Difficulty paying attention or concentrating. Indecisiveness. Impaired memory.	Easy fatigue. Insomnia/hypersomnia.
